# A prospective randomized comparative study to evaluate the effect of palliative hypo-fractionated radiotherapy with concurrent chemotherapy versus hypo-fractionated radiotherapy alone in advanced and unresectable head and neck cancer with no metastasis

**DOI:** 10.3332/ecancer.2023.1541

**Published:** 2023-04-28

**Authors:** Aditya Kumar Uttam, Arun Kumar Yadav, Shreya Jalota, Renu Singh, Shivani Malik, Ashok Kumar Arya

**Affiliations:** Sarojini Naidu Medical College, Agra 282003, Uttar Pradesh, India

**Keywords:** hypo-fractionated radiotherapy, locally advance unresectable head and neck cancer, palliative radiotherapy, pain control

## Abstract

**Introduction:**

A short duration, palliative radiotherapy schedule for locally advanced and unresectable head and neck cancer (LAUHNC) was evaluated in terms of palliation of cancer-related symptoms and acute toxicities.

**Aims and objectives:**

The aim of the study was to compare the role and feasibility of hypo-fractionated radiotherapy with concurrent chemotherapy and hypo-fractionated radiotherapy in LAUHNC.

**Materials and methods:**

All the patients included in this study of LAUHNC were not fit for curative treatment. These patients are assessed on the basis of quality of life (QOL), tumour response, toxicities, and relief in symptoms. QOL was assessed on the basis of University of Washington QOL questionnaire version 4 before and after treatment. Patients are divided into two arms, Arm A patients received 40 Gy in ten fractions concurrent cisplatin 50 mg/m2 with weekly and Arm B patients received 40 Gy in ten fractions. To assess the tumour response response evaluation criteria in solid tumours criteria were used.

**Results:**

A total of 40 patients were enrolled in this study, with 20 patents in both arms. Three patients defaulted during treatment and one patient died during treatment. A total of 36 patients completed treatment. Before treatment common complaints was distressing pain in primary site, and difficulty in chewing and swallowing. After treatment there was reduction of pain and improvement in swallowing in both arms. Overall QOL improvement in Arm A (28.89 ± 18.44 to 46.67 ± 15.34) and in Arm B (31.11 ± 15.68 to 43.33 ± 15.72). Neither of the arms experienced grade IV mucositis and skin reaction.

**Conclusion:**

Toxicity in the form of mucositis and dermatitis was higher in the concurrent hypo-fractioned arm compared to the only hypo-fractionated radiotherapy arm during the treatment and in follow up period. The QOL in both individual arms showed statistically significant results, however when the QOL of both the arms was compared, the results were not statistically significant.

## Introduction

In 2020, it was estimated that about 65,630 new cases of oral cavity, pharyngeal, and laryngeal cancer will occur which account for 3.6% of new cancer cases in the United States, as an estimated 14,500 deaths from head and neck cancers will occur during the same period of time [1]. Oral cancer is the most common cancer in India amongst men (16.1% of all cancers) and the fourth most frequent cancer among Indian women [2]. India has one-third of oral cancer cases in the world [3]. Oral cancer accounts for around 30% of all cancers in India [4]. The majority of the head and neck cancers present in locally advanced stages (stages III and IV). Lack of knowledge among the population, socio-economic restraints, and limited availability of medical care to the susceptible population contribute to the higher incidence of locally advanced HNSCC.

Patients often present with severe symptoms and there are no evidence-based guidelines for standard practice of palliative care in advanced head and neck cancer [5, 6]. Surgery is typically not an option for patients with advanced lesions and poor performance status because a sizeable amount of disease would still be present. Concomitant chemoradiotherapy improves locoregional control and overall survival (OS) in locally advanced head and neck cancer (LAHNC) compared with radiotherapy (RT) alone; consequently, chemoradiation is the standard of care for these patients [7]. There is a dearth of such information on advanced HNSCC, despite the existence of significant evidence regarding the advantages of palliative hypo-fractionated RT in patients with advanced solid tumours. It is challenging to provide palliation on time in developing nations due to staffing and equipment constraints. A short course of high dose radiation (hypo-fractionation) can downsize the bulk of the tumour within a short span of time, resulting in good symptom relief with minimal residual toxicity. Altered fractionation is an alternative for patients who are not suitable for concurrent RT and can improve OS compared with conventional fractionation RT (CFRT – 35, 2-Gy fractions over 7 weeks) alone [8, 9]. Accelerated RT, in which the total dose is delivered in a short period of time, has radiobiological advantages and is also associated with improved clinical outcomes [10, 11]. Hypo-fractionation is an attractive method for accelerating RT and has been used with success in other tumour sites, showing comparable outcomes and a reduced cost compared to those of CFRT [12–15].

In this study, we assessed the quality of life (QOL) of locally advanced unresectable head and neck cancer using 2D RT with cobalt-60 machine before and after hypo-fractionated RT with or without use of chemotherapy, objective response to tumour and nodal site and comparing toxicities due to addition of chemotherapy.

## Material and methods

This prospective study was conducted on patients who reported to the RT department. All patients and family were informed about the stages of disease, prognosis, palliative intent and written consent was taken. Duration of the study was approximately 18 months starting from 2020 to 2022. All patients were at unresectable stage IV with no metastasis head and neck cancer and biopsy proven. In this prospective study, 40 patients were included and these patients were divided into two arms through the simple 1:1 randomization method. Patients who did not give consent for study and patients who had received prior definitive treatment in the form of RT or chemotherapy were excluded from the study.

Distressing presenting symptoms like pain, painful ulcers, dysphagia, ondynophagia, breathing difficulty and neck swelling were recorded and graded according to the Washington QOL version 4.0 as per the patient’s reporting of the complaint. Dental evaluation was done prior to RT. Patients were also managed symptomatically according to their complaints.

All patients in Arm A received 40 Gy in ten fractions with two fractions per week (Tuesdays and Fridays). Patients were immobilized in the supine position. A primary and nodal gross tumour volume was outlined based on clinical examination. RT portal included gross tumour volume with 2 cm margin and the high risk nodal regions. The biologically effective dose for this regimen is 56 Gy for tumour tissue and 93.3 Gy for late reacting tissue. In the telecobalt machine (theratron Phoenix), RT was administered using the parallel opposed lateral approach [6]. In Arm B weekly injection cisplatin 50 mg/m^2^ was given every Tuesday with the same RT protocol.

### Assessment of toxicity and QOL

Patients were assessed weekly for assessment of treatment-related toxicity and tolerance. Treatment-related toxicity including radiation mucositis and dermatitis was assessed using the radiation therapy oncology group criteria [17]. Patients were explicitly questioned about the difference in symptom alleviation from their initial presentation. The University of Washington QOL Questionnaire (UW-QOL) version 4.0 was used to assess the subjective symptoms and QOL before and after 2 weeks of RT. It consists of three general questions with discrete ordinal responses and 12 domains. Zero denotes the worst possible answer and 100 the greatest [18]. Utilizing the response evaluation criteria in solid tumours criteria, the tumour response was evaluated.

### Post-treatment follow up

Tumour response and symptom relief were periodically monitored at 1 month intervals for 1 year and then 3 monthly or earlier if necessary. Patients with progressive disease were treated with symptomatic care and were initiated with injection methotrexate 50 mg weekly and tyrosine kinase inhibitor (Tablet Geftinib 250 mg once daily).

### Statistical analysis

The IBM SPSS Statistics 29.0 program was used to do the statistical analysis. Using descriptive statistics, patient demographic profiles, treatment variables, and radiation-induced toxicity were examined. The paired *t-*test was used to evaluate the UW-QOL questionnaire. The paired *t*-test was used to evaluate the UW-QOL questionnaire. *p* < 0.05 was considered as statistically significant.

## Results

A total of 40 patients were included in the study out of which 3 defaulted and 1 patient died during treatment. Twenty nine patients completed treatment in 5 weeks and 7 patients completed their treatment in a median time of 7 weeks, during treatment 13 patients required nasogastric tube and 7 patients were prescribed opioid analgesic before and during treatment and 2 patients were prescribed to continue on opioid post-treatment for severe pain, the remaining patients had mild-to-moderate pain. Patients achieved a BED of 56 Gy to the tumour.

The demographic profile is described in Table 1. Male predominance was observed (80.6%) and the median age was 48 (25–72) years. The most common site was oral cavity and the most frequent complaint was pain followed by dysphagia. Both arms of patients tolerated the treatment well. No grade IV mucosal and skin toxicity was seen in either arm during treatment. Grade III mucosal toxicity was seen in five patients and two patients in Arm A and Arm B, respectively, and in Arm A two patients and in Arm B no patient experienced grade III skin toxicity. No patient died due to acute toxicity. One patient died due to a non-oncological reason. Seventy nine percent of patients completed treatment without any break. During RT nine patients were hospitalized for mucositis management and they were kept on parental nutritional support.

Patients were assessed on the basis of the Karnofsky Performance Scale (KPS) before and 1 month after completion of treatment, there was significant improvement in both the arms before and after treatment (Arm A (63.89 ± 5.02 to 76.11 ± 6.08) *p*-value < 0.0001, Arm B (63.33 ± 4.85 to 74.44 ± 7.05) *p*-value < 0.0001)) as depicted in Table 2.

Using UW-QOL questionnaire v4, the mean ± standard deviation before treatment and after 1 month of completion of treatment in both the arms, patients were assessed (Table 3). In overall QOL, there was significant improvement in both the arms (Arm A (28.89 ± 18.44 to 46.67 ± 15.34) *p* value < 0.001 Arm B (31.11 ± 15.68 to 43.33 ± 15.72) *p* value < 0.001)). There was significant improvement in pain, swallowing, appearance and activity but significantly the score decreased in taste, saliva, mood and anxiety in both the arms.

After 1 month of completion of treatment an objective response assessment was done on both tumour and node. There was no complete response in either arm. Most patients presented with partial tumour and nodal response in both the arms. While 14 patients (77.8%) and 13 patients (72.2%) had partial response and 4 patients (22.2%) and 5 patients (27.8%) had stable response in Arm A and Arm B, respectively.

While after 1 month of follow up in both arms 1 (5.6%) patient had progressive nodal disease, 13 patients (72.2%) and 11 patients (61.1%) had partial nodal response and 4 patients (22.2%) and 6 patients (33.3%) had stable disease, respectively. As shown in Table 3, when we compare overall QOL in Arm A and Arm B the comparison was statistically insignificant (*p* value = 0.5232).

## Discussion

The main aim of this study was palliation of distressing symptoms like painful ulcers, pain, difficulty in swallowing and to study treatment-related toxicities and QOL. In our study, improvement in QOL and cost benefit issue are important because our patients belonged to a lower socioeconomic status and came to us beyond the incurable stage. Radical treatment in the form of an aggressive multi-modality approach is not successful in all of these patients because of poor performance status and unresectability. These advanced and unresectable cases need palliative treatment and/or best supportive care.

### Radiation toxicity and relief of symptoms

A similar RT regimen like the one in this study was investigated by Mudgal *et al* [19]. Grade III acute mucosal and skin toxicities were seen in 8.6% and 2.17% cases, respectively. There was no Grade IV mucosal or skin toxicity seen during the treatment. 6.5% patients required nasogastric tube feeding due to progressive difficulty in swallowing. All the patients completed the entire treatment.

The toxicity profile with concurrent carboplatin given with hypo-fractionated accelerated RT was shown by Madhava *et al* [20] Grade 3 mucositis persisting for 4 weeks was observed in two patients and Grade 3 mucositis lasting 4 weeks or more in three patients, but two of them received AUC 5 carboplatin. ‘Hypo Trial’ Porceddu *et al* [21] in his study concluded that Grade 2 and 3 mucositis were reported in 37% and 26%, respectively, all of which had resolved by 4 weeks post-treatment. Grade 3 skin reaction was reported in 11% patients. In present study, concurrent hypo-fractionated RT regimen mucositis increased during half (5#) treatment in compare with hypo-fractionated RT regimen. No grade III and grade IV mucositis and dermatitis seen during treatment, in Arm A four patients experienced no mucositis nine patients grade I mucositis and five patients grade II mucositis on the other hand Arm B, six patients experienced no toxicity and eight patients grade I mucositis and four patients grade II mucositis.

At the end of the complete treatment, in Arm A 13 patients had grade II mucositis and 5 had grade III mucositis. Whereas in Arm B 16 patients had grade II mucositis, 2 had grade III mucositis.

After 1 month of follow up mucositis decreased in both the arms but decreased more in the hypo-fractionated RT arm compared to the concurrent hypo-fractionated RT arm. Neither arm had any grade III or IV mucositis. One month after treatment completion, in Arm B all patients had grade I mucositis or no mucositis. However, in Arm A three patients did show grade II mucosal toxicity.

In Arm A and B, during RT dermatitis increased due to high dose per fraction, after completion of 5# neither arm has grade III and IV dermal toxicity. After completion of treatment 10 # Arm A dermal toxicity was slightly more compared to Arm B, two patients experienced grade III toxicity in Arm A but no patients reported grade III toxicity in Arm A. After 1 month follow up hyper pigmentation seen in both arms, 11 and 13 patients reported no toxicity and 7 and 5 patients reported grade I dermal toxicity in Arm A and B, respectively.

### QOL analysis

Porceddua *et al* [21] ‘Hypo Trial’ conducted a trial to assess the rate of tumour response to a hypo-fractionated course of RT of 30 Gy in five fractions at 2/week, at least 3 days apart, with an additional boost of 6 Gy for small volume disease (<3 cm) in suitable patients and QOL calculated on the basis of WHO performance status reported energy levels improved in 29% and did not change in 24% while 76% experienced an improvement in the ability to work. Sixty-seven percent reported an improvement in overall pain and 76% an improvement in pain in the mouth, throat or neck. With respect to the ability to eat solid foods 33% reported an improvement, 52% no change and 14% a deterioration.

Das *et al* [16] conducted a study on patients who received 40 Gy in ten fractions. QOL assessment showed improvement in social well-being (17.4 versus 20.01, *p* = 0.03), but no significant change was observed in head and neck specific score (25. I versus 25.0, *p* = NS) after treatment. Reduction of pain was observed in 88% patients and 60% patients had improvement of performance status.

In 2019, Mudgal *et al* [19] conducted a study on patients with advanced (stage IV) head and neck cancers. All patients received 40 Gy in ten fractions with two fraction/week. A total of 50 patients were enrolled in the study, out of which 46 completed the planned treatment of 40 Gy in 10 fractions. Statistically significant improvements were observed in overall QOL (26.9 ± 9.65 to 55.65 ± 19.28).

It is known till now that no studies have been conducted on QOL improvement in concurrent hypo-fractionated palliative RT.

In the present study, the main presenting complaints of patients were pain and dysphagia, 70% of patients presented with two or more distressing symptoms. Using UW-QOL questionnaire v4, the mean ± standard deviation before treatment and after 1 month of completion of treatment in both the arms, patients were assessed. There was pain relief in Arm A from 37.5 ± 19.65 to 54.17 ± 12.86 pre-treatment and post-treatment (*p* = 0.002*) and in Arm B, 38.89 ± 17.62 to 51.39 ± 13.48 pre-treatment and post-treatment (*p* = 0.006*) but when compared to Arm A the Arm B result was insignificant (*p* = 0.5309). In Arm A swallowing improved from 33.00 ± 19.60 to 44.00 ± 19.60 pre-treatment and post-treatment (*p* = 0.111) and in Arm B swallowing improved from 14.71 ± 23.48 to 26.47 ± 25.72 (*p* = 0.041*) and when compared to Arm A, Arm B result was statically significant. In overall QOL, there was significant improvement in both the arms (Arm A (28.89 ± 18.44 to 46.67 ± 15.34) *p* value <0.001 Arm B (31.11 ± 15.68 to 43.33 ± 15.72) *p* value < 0.001)) but when compared Arm A and Arm B side by side the result was insignificant (*p* = 0.4714). There was significant improvement in pain, swallowing, appearance and activity but significantly the score decreased in taste, saliva, mood and anxiety in both the arms.

### Nodal and tumour response analysis

Mudgal *et al* [19] conducted a study which concluded that good objective response was seen in 82.6% and 84.7% of patients at primary and nodal sites, respectively. In the post-treatment evaluation, 6.5% patients showed a complete response at primary and 13% at nodal site, 76.1% and 77.1% patients showed partial response at primary and nodal sites, respectively. 17.3% and 15.2% patients had stable disease at primary and nodal sites, respectively. While Madhava *et al* [20] concluded that a total of 84% had complete response to chemo radiation. One patient 5% was classified as a partial response and achieved complete response after planned post-chemo RT neck dissection. 11% had persistent primary disease after chemo radiation. And Jacinto *et al* [22] concluded in his study the overall rate of response tumour and nodal both were 95% after 2 months. The rate of complete response of the primary site was 85%. The nodal complete response was 40%. The overall complete response was 85% for patients with resectable disease and 35%for patients deemed unresectable.

In this study, WHO response assessment was done at 4 weeks, no patient had progression in the primary tumour in either of the 2 arms, while 14 patients (77.8%) and 13 patients (72.2%) has partial response and 4 patients (22.2%) and 5 patients (27.8%) has stable response in Arm A and Arm B, respectively. While after 1 month of follow up in both arms 1 (5.6%) patient has progressive nodal disease, 13 patients (72.2%) and 11 patients (61.1%) has partial nodal response and 4 patients (22.2%) and 6 patients (33.3%) has stable disease, respectively. (Table 4).

## Conclusion

In the present study, hypo-fractionated palliative RT showed promising results in LAHNC in both the arms with high dose rate at tumour site, good symptomatic relief and minimal toxicity. However, the addition of concurrent chemotherapy to the hypo-fractionated regimen showed a higher rate of mucositis and dermatitis and similar QOL profile, but conferred no statistically significant advantage in terms of tumour response. The limitation of this current study is a shorter follow-up period and small sample size.

## List of abbreviations

CFRT: Conventional fractionation radiotherapy; CTRT: Concurrent chemo radiotherapy; KPS: Karnofsky Performance Scale; LAUHNC: Locally advanced and unresectable head and neck cancer; SCC: Squamous cell carcinoma; UW-QOL: University of Washington quality of life questionnaire.

## Conflicts of interest

The author(s) declare that they have no conflict of interest either financial or non-financial.

## Funding

No direct or indirect funding was received for this clinical work.

## Ethics board approval

SNMC/IEC/THESIS/2022/144.

## Figures and Tables

**Table 1.** Demographic profile.

Particulars		Distribution
Age	Range	25–72 years
	Median	48 years
Gender	Male	29 (80.6%)
	Female	7 (19.4%)
Habits	Tobacco	31 (86.1%)
	Smoking	22 (61.1%)
	Alcohol	19 (52.8%)
Histo-pathology report	Well differentiated squamous cell carcinoma (SCC)	7 (19.4%)
	Moderately differentiated SCC	23 (63.9%)
	Poorly differentiated SCC	4 (11.1%)
	Undifferentiated-SCC	2 (5.6%)

**Table 2.** Pre-treatment and post-treatment KPS score evaluation in concurrent hypo-fractionated and hypo-fractionated arm.

KPS score	With CTRT		Only RT	
	**Pre-treatment**	**Post-treatment**	**Pre-treatment**	**Post-treatment**
60	11 (61.11%)	1 (5.56%)	12 (66.67%)	2 (11.11%)
70	7 (38.89%)	5 (27.78%)	6 (33.33%)	6 (33.33%)
80	0	12 (66.67%)	0	10 (55.56%)
Total patients	18	18	18	18
Mean	63.89 ± 5.02	76.11 ± 6.08	63.33 ± 4.85	74.44 ± 7.05
*p* value	0.0001*		0.0001*	

* Statistically significant (*p*-value < 0.05)

**Table 3.** Pre-treatment and post-treatment QOL assessment in concurrent hypo-fractionated and hypo-fractionated arm.

Particulars	With CTRT			Only RT			p-value
	Pre-treatment	Post-treatment	p-value	Pre-treatment	Post-treatment	p-value	
Pain	37.5 ± 19.65	54.17 ± 12.86	0.002*	38.89 ± 17.62	51.39 ± 13.48	0.006*	0.5309
Appearance	37.50 ± 21.44	48.61 ± 18.13	0.028*	40.28 ± 15.19	44.44 ± 16.17	0.330	0.4714
Activity	52.78 ± 14.57	62.50 ± 12.86	0.030*	54.17 ± 17.68	61.11 ± 15.39	0.020*	0.7705
Recreation	29.41 ± 13.21	41.67 ± 19.17	0.002*	22.22 ± 16.91	33.33 ± 22.69	0.072	0.2418
Swallowing	33.00 ± 19.60	44.00 ± 19.60	0.111	14.71 ± 23.48	26.47 ± 25.72	0.041*	0.0277*
Chewing	13.89 ± 23.04	25.00 ± 25.72	0.0418*	11.11 ± 21.39	19.44 ± 25.08	0.187	0.9926
Speech	27.50 ± 23.33	42.17 ± 22.08	0.0416*	29.33 ± 22.32	42.17 ± 22.08	0.049*	1.0000
Taste	86.78 ± 17.06	38.50 ± 23.33	0.001*	89.33 ± 23.66	44.00 ± 22.64	0.001*	0.4778
Saliva	86.83 ± 20.51	45.83 ± 16.55	0.001*	83.06 ± 20.88	44.00 ± 19.60	0.001	0.7640
Shoulder	98.11 ± 8.01	98.11 ± 8.01	1.00	98.11 ± 8.01	98.11 ± 8.01	1.00	1.00
Mood	31.94 ± 11.52	18.06 ± 16.73	0.037*	29.17 ± 17.68	16.67 ± 14.85	0.095	0.7937
Physical Domain	27.78 ± 20.81	40.28 ± 21.25	0.001*	30.56 ± 20.21	38.89 ± 19.60	0.009*	0.9978
HRQOL in last 7 days	26.67 ± 15.34	37.78 ± 13.53	0.001*	27.78 ± 13.96	35.56 ± 16.17	0.004*	0.6579
Overall QOL	28.89 ± 18.44	46.67 ± 15.34	0.001*	31.11 ± 15.68	43.33 ± 15.72	0.001*	0.5232

* Statistically significant (*p*-value < 0.05)

**Table 4.** Objective response assessment.

Response	Tumor response		Nodal response	
	CTRT	RT	CTRT	RT
Progressive disease	0	0	1 (5.6%)	1 (5.6%)
Partial response	14 (77.8%)	13 (72.2%)	13(72.2%)	11 (61.1%)
Stable disease	4 (22.2%)	5 (27.8%)	4 (22.2%)	6 (33.3%)
Total patients	18	18	18	18
